# High-intensity leg cycling alters the molecular response to resistance exercise in the arm muscles

**DOI:** 10.1038/s41598-021-85733-1

**Published:** 2021-03-19

**Authors:** Marcus Moberg, William Apró, Igor Cervenka, Björn Ekblom, Gerrit van Hall, Hans-Christer Holmberg, Jorge L. Ruas, Eva Blomstrand

**Affiliations:** 1 Department of Physiology, Nutrition and Biomechanics, Swedish School of Sport and Health Sciences, Stockholm, Sweden; 2 Department of Clinical Science, Intervention and Technology, Karolinska Institutet, Stockholm, Sweden; 3 Department of Physiology and Pharmacology, Karolinska Institutet, Stockholm, Sweden; 4 Department of Biomedical Sciences, Faculty of Health and Medical Sciences, University of Copenhagen, Copenhagen, Denmark; 5 Clinical Metabolomics Core Facility, Clinical Biochemistry, Rigshospitalet, Copenhagen, Denmark; 6 The Swedish School of Sport and Health Sciences, Box 5626, 114 86 Stockholm, Sweden

**Keywords:** Metabolism, Proteins

## Abstract

This study examined acute molecular responses to concurrent exercise involving different muscles. Eight men participated in a randomized crossover-trial with two sessions, one where they performed interval cycling followed by upper body resistance exercise (ER-Arm), and one with upper body resistance exercise only (R-Arm). Biopsies were taken from the triceps prior to and immediately, 90- and 180-min following exercise. Immediately after resistance exercise, the elevation in S6K1 activity was smaller and the 4E-BP1:eIF4E interaction greater in ER-Arm, but this acute attenuation disappeared during recovery. The protein synthetic rate in triceps was greater following exercise than at rest, with no difference between trials. The level of PGC-1α1 mRNA increased to greater extent in ER-Arm than R-Arm after 90 min of recovery, as was PGC-1α4 mRNA after both 90 and 180 min. Levels of MuRF-1 mRNA was unchanged in R-Arm, but elevated during recovery in ER-Arm, whereas MAFbx mRNA levels increased slightly in both trials. RNA sequencing in a subgroup of subjects revealed 862 differently expressed genes with ER-Arm versus R-Arm during recovery. These findings suggest that leg cycling prior to arm resistance exercise causes systemic changes that potentiate induction of specific genes in the triceps, without compromising the anabolic response.

## Introduction

Skeletal muscles possess a unique capacity to adapt to diverse exercise stimuli. Resistance training stimulates protein synthesis^1,2^ and accretion^3^ mainly via activation of the mammalian target of rapamycin complex 1 (mTORC1) pathway. While mTORC1 activation has been shown following acute endurance exercise^4,5^, the adaptation generally associated with endurance training, enhanced oxidative capacity^6^, mainly occurs through repeated stimulation of mitochondrial gene expression, a process in which the transcription factor peroxisome proliferator-activated receptor gamma co-activator 1α (PGC-1α1) is believed to play a particularly important role^7–9^. PGC-1α1 is one of several coactivator variants expressed by the PGC-1α gene in skeletal muscle^10^. Another coactivator variant relevant to this study is PGC-1α4, which has been linked to the regulation of muscle mass^11^, but in human muscle has been shown to be expressed following both resistance and endurance exercise^12,13^.

These two types of exercise are commonly combined by athletes and recreationally active individuals, and may be beneficial for endurance performance^14^. In contrast, some studies have shown that when the two exercises are combined, they may impair strength training adaptations, although this is not a general finding^15–18^ However, suboptimal resistance exercise performance due to residual fatigue following endurance exercise^19^, could influence both acute molecular responses and adaptation to training. In addition, acute concurrent exercise has been shown to induce a greater expression of genes and proteins involved in proteolysis compared to resistance exercise alone^20^, which could influence long term muscle adaptations. Concurrent exercise with different sets of muscles when performed in the same session, such as lower-body endurance and upper-body resistance exercise, may thus be more appropriate when applicable. While a large number of studies have investigated the molecular response to different forms of concurrent exercise in the same muscle group^18,20^, including the triceps muscle^21^, no previous study has investigated the effect of separating the exercise modes between different muscle groups.

Although the molecular responses to such a combination have yet to be characterized, some evidence indicates that changes in systemic factors influence muscular adaptation to training. For example, Widegren et al.^22^ found a pronounced increase in the phosphorylation of p38 mitogen-activated protein kinase (p38 MAPK) and cyclic AMP response element binding protein (CREB) in the non-exercised leg following unilateral cycling. Furthermore, unilateral resistance as well as endurance exercise elevated mTORC1 signaling in both resting and exercising leg^4,23^. An increasing number of studies have reported the existence of contraction induced factors (e.g. peptides, miRNA and metabolites) that are released from the muscle, alone or embedded in extracellular vesicles, which can exert crosstalk to non-exercised tissue^24,25^. While the relevance of altered signaling responses in resting muscle is unclear, the potential molecular crosstalk between two different active muscle groups may be significant; when a muscle is activated and triggered for adaptation, the impact of a preceding systemic release of so called exerkines might be enhanced as compared to the effect on a resting muscle.

Given the lack of knowledge regarding the potential molecular crosstalk between two muscle groups performing different modes of exercise and, the practical relevance of performing this type of concurrent exercise, the present study was designed to elucidate the effects of concurrent exercise, involving different muscle groups, on the molecular responses. To this end, our subjects took part in a randomized crossover trial consisting of two different sessions. In one session they performed high intensity interval exercise with the lower body followed by resistance exercise with their arms. In the other session they performed only the arm resistance exercise protocol. Triceps muscle biopsies during subsequent recovery were used for analysis of fractional rate of protein synthesis, mTORC1 signaling, mRNA levels for relevant ubiquitin ligases, PGC-1α1, and PGC-1α4 as well as for global gene expression analysis by RNA-sequencing. Moreover, changes in plasma levels of hormones and muscle levels of glycogen and lactate were determined. Our main research question was to examine if cycling with the lower extremities prior to resistance exercise involving the triceps muscle would enhance the exercise-induced increase in mTOR signaling, rate of protein synthesis, as well as the expression of catabolic genes in this muscle.

## Methods

### Subjects

The eight healthy, training accustomed male subjects were all required to be free from injury and medical conditions as well as have performed resistance exercise involving the arms and legs two or three times each week, plus endurance exercise on a regular basis for at least the preceding six months. In addition, our subjects were required to demonstrate a 10-repetition maximum (10RM) with seated arm extensions equal to at least 125% of their body weight (mean 113 ± 4 kg), as well as a minimum peak oxygen uptake (VO_2 peak_) on the cycle ergometer of ≥ 50 ml min^−1^ kg^−1^ (mean 55 ± 5). They were 31 ± 5 years of age, 182 ± 5 cm tall and weighed 80 ± 5 kg. After being informed of the purpose of the study and associated risks, all subjects gave their written informed consent to participate. This study was pre-approved by the Regional Ethical Review Board in Stockholm (Dnr 2011/697-31/2) and performed in accordance with the principles outlined in the Declaration of Helsinki.

### General design

**Figure 1:**
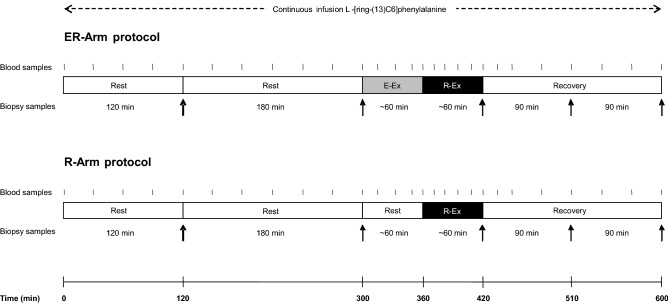
Schematic overview of the experimental protocol. The black arrows denote time points at which triceps brachii biopsies were taken and the small vertical black lines when blood was drawn. E-Ex: the high-intensity interval cycling; R-Ex: the resistance exercise involving seated arm extensions.

The hypothesis was tested employing a randomized cross-over design in which each subject performed one session of high-intensity interval cycling followed by upper-body resistance exercise (ER-Arm) and another session of resistance exercise only (R-Arm). These two sessions, separated by 14–16 days, are illustrated schematically in Fig. 1.

The subjects were instructed to maintain their habitual dietary intake and physical activity throughout the experimental period, with the exception that they were told to refrain from physical exercise during the two days prior to each trial. In addition, they were instructed to record their food intake during two days before the first trial and to repeat this same diet for the two days prior to the second trial.

### Preliminary tests

Three weeks before the main experiments, preliminary tests on a mechanically braked cycle ergometer (Monark 839E, Vansbro, Sweden) were performed. Oxygen uptake at four or five submaximal work rates as well as peak oxygen uptake (VO_2peak_) were determined utilizing an on-line system (Oxycon Pro, Erich Jaeger GmbH, Hoechberg, Germany). The subjects exercised at a pedalling rate of 80 rpm and heart rate (HR) was recorded continuously (Polar Electro OY, Kempele, Finland). From these measurements, the work rate corresponding to 85% of VO_2peak_ was calculated for each individual.

Following determination of peak oxygen uptake, each subject rested for 20 min before assessment of the two-armed 10RM seated on an arm extension machine (131SEC, Nordic Gym, Bollnäs, Sweden). Three sets of 10 repetitions each at 0, 25 and 75% of body weight were followed by maximal effort at 125% body mass. If the subjects could perform 10 repetitions or more at the highest load, the load was increased gradually until they failed to perform 10 acceptable repetitions. All repetitions involved an elbow angle of 75°–180° and all sets at ≥ 125% body mass were separated by a 5-min rest.

On two other days, the subjects carried out two sessions of familiarization one week apart, in order to minimize training effects during the experiments themselves. These sessions consisted of the ER-Arm protocol (see below), with the same intensity of cycling and load of arm extension exercise as in the experimental exercise protocol. The specific purpose was to allow the subjects to become accustomed to performing the high-volume and fatiguing resistance exercise following a demanding bout of endurance exercise.

### Experimental protocol

Following an overnight fast starting at 9.00 PM the evening before, the subjects arrived in the laboratory on days of experimentation at 5.30 AM. 17G Teflon catheters were inserted into the antecubital vein of both arms, one for repeated blood sampling and the other for continuous tracer infusion. Following collection of a baseline blood sample, a primed constant infusion of L-[ring-13C6]-phenylalanine (2 µmol kg^−1^ followed by 0.05 µmol kg^−1^ min^−1^, Cambridge Isotope Laboratories, Danvers, MA, USA) was initiated and maintained for the duration of the experiment (~ 10 h). Following two hours of tracer infusion, the first resting biopsy was collected under local anaesthesia from the long head of one triceps brachii utilizing a Weil-Blakesley conchotome (AB Wisex, Mölndal, Sweden) as described by Henriksson^26^. Three hours later, a second resting biopsy was collected to allow calculation of the rate of mixed muscle protein synthesis at rest. During these initial five hours of rest and tracer infusion, blood samples were collected every 30 min into EDTA-tubes.

For the ER-Arm trial, the endurance session began with a 15-min warm-up on the cycle ergometer (5 min at 50 W and 10 min at 100 W), followed by five 4-min intervals at a work rate corresponding to 83 ± 3% of VO_2peak_. These high-intensity intervals were separated by 3 min of low-intensity cycling at 100 W. Blood samples were drawn after the warm-up, after the third and last interval. The subjects then cycled for an additional 10 min at 100 W, followed by five minutes of rest.

Thereafter, the subjects were seated in the arm extension machine and performed 3 warm-up sets of 10 repetitions each at 25%, 50% and 75% of their 10RM separated by 3 min and then 10 sets of heavy resistance exercise. The initial load was their 10RM, with a gradual decrease that allowed the subjects to continue performing 9–12 repetitions until final fatigue. At this point during the last 2 sets, the load was lowered by 10 kg and the subjects immediately performed 5 additional repetitions. The sets were separated by 3 min of recovery while seated.

Blood was collected in EDTA tubes prior to and after the warm-up, as well as after the fourth, seventh and final tenth set. Each subject performed the same number of repetitions, with matching workload and time under tension during the resistance exercise in both trials. In the R-Arm trial, cycling was replaced by supine rest with blood sampling at the same time-points as in the ER-Arm trial.

Within 30 s after completion of the last set of resistance exercise in both trials, a third muscle biopsy was taken with the subject still seated in the exercise machine. Thereafter, two additional biopsies were taken after 90 and 180 min of recovery in the supine position. The biopsies were taken alternately from the right and left arms, starting with the right at the first session. Accordingly, biopsy number 1, 3, and 5 was taken in the right arm and number 2 and 4 in the left arm during the first session. During the second session the biopsies were initiated in the left arm, which thus had three biopsies in that session. The first biopsy was taken approximately 12 cm from the olecranon and the subsequent ones 2.5 cm proximal or slightly medial to the previous one in the first and second trials, respectively. Biopsies were immediately blotted free of blood and frozen in liquid nitrogen and subsequently stored at − 80 °C for later analysis. Blood samples were drawn after 15, 30, 60, 90, 120, 150 and 180 min of recovery, at which time the infusion was terminated.

### Plasma analyses

Blood samples (4 ml) were kept on ice for no more than a few minutes and then centrifuged at 10,000×*g* at 4 °C for 3 min and the plasma stored at -80 °C. The glucose concentration was determined with a Biosen C-Line (EKF Diagnostics, Cardiff, UK) and lactate analyzed spectrophotometrically as described by Bergmeyer^27^. Plasma cortisol and insulin levels were determined using an ELISA kit (Calbiotech, Spring Valley, CA, USA; Mercodia, Uppsala, Sweden) in accordance with the manufacturer’s instructions.

### Muscle tissue processing

The muscle tissue was lyophilized and dissected thoroughly free of blood and connective tissue under a light microscope (Carl Zeiss Microscopy, Jena, Germany), leaving only very small intact bundles of fibers, which were carefully mixed together and then divided into aliquots for subsequent analysis.

### Muscle glycogen and lactate

Muscle glycogen in the supernatant and pellet of the immunoprecipitated muscle homogenate (see below) was determined according to Leighton et al. (1989). The glycogen concentration is presented as the sum of these measurements. Lactate was measured in TCA extracts of muscle (40 µl mg^−1^) after neutralizing the sample with 1 M KOH. The concentration was determined spectrophotometrically as described by Bergmeyer^27^. Muscle level of lactate were determined in seven subjects due to limited amount of muscle tissue from one subject.

### Analysis of stable isotope enrichment

The procedure for assessment of L-[ring-13C6]-phenylalanine enrichment has been described previously^20^. In brief, enrichment in both the plasma and intracellular muscle pool was determined by gas chromatography–tandem mass spectrometry (GC–MS/MS, Tracer GC Ultra-TSQ Quantum; Thermo Scientific, Palo Alto, CA, USA) with electron impact ionization and selective ion monitoring for 336, 342, and 345 m/z, after derivatization with N-methyl-N-(tert-butyldimethylsilyl)-trifluoroacetamide. To quantify protein-bound enrichment, amino acids purified from 5 mg lyophilized muscle were first converted to their N-acetyl-n-propyl amino acid esters and then analyzed by gas chromatography–combustion–isotope ratio mass spectrometry (GC–C–IRMS, Hewlett Packard 5890-Finnigan GC combustion III-Finnigan Deltaplus; Finnigan MAT, Bremen, Germany).

### Calculations of the fractional synthetic rate of mixed muscle protein

The fractional synthetic rate of mixed muscle protein was calculated employing the standard precursor-product approach:

$${\text{FSR }} = \Delta E_{{p\;{\text{phe}}}} / \, \left( {E_{{ic\;{\text{phe}}}} \times {\text{ T}}} \right) \, \times { 1}00$$

where Δ*E*_*p* phe_ is the difference in protein-bound phenylalanine enrichment between two biopsies; *E*_*ic* phe_ the average intracellular phenylalanine enrichment in these two biopsies or the average plasma enrichment during the corresponding periods; and T length of tracer incorporation in hours, multiplied by 100 in order to obtain FSR in percentage per hour (% × h^−1^).

### Immunoblotting

Samples (approx. 4 mg) of lyophilized and dissected muscle tissue were homogenized in ice-cold buffer (100 µl mg^−1^ dry weight) containing 2 mM HEPES (pH 7.4), 1 mM EDTA, 5 mM EGTA, 10 mM MgCl_2_, 50 mM β-glycerophosphate, 1% TritonX-100, 1 mM Na_3_VO_4_, 2 mM dithiothreitol, 1% phosphatase inhibitor cocktail (Sigma P-2850) and 1% (v/v) Halt Protease Inhibitor Cocktail (Thermo Fischer Scientific, Rockford, IL, USA) using a BulletBlender (Next Advance, Troy, NY, USA). The homogenates thus obtained were rotated for 30 min at 4 °C and centrifuged at 10,000×*g* for 10 min at 4 °C to remove myofibrillar and connective tissue debris and the resulting supernatant collected.

The protein concentrations of the supernatants (diluted 1:10 with distilled water) were determined by the Pierce 660 nm protein assay (Thermo Scientific). Appropriate aliquots were then diluted in Laemmli sample buffer (LSB) (Bio-Rad Laboratories, Richmond, CA, USA) and homogenizing buffer to obtain a final protein concentration of 1.0 µg µl^−1^; heated at 95 °C for 5 min to denature the proteins, and subsequently stored at − 20 °C until separation on SDS-Page.

For separation, 20 µg protein from each sample was loaded onto Criterion TGX gradient gels (4–20% acrylamide; Bio-Rad Laboratories) and electrophoresis performed on ice at 300 V for 30 min. Next, these gels were equilibrated in transfer buffer (25 mM Tris base, 192 mM glycine and 10% methanol) for 30 min at 4 °C, following which the proteins were transferred to polyvinylidine fluoride membranes (Bio-Rad Laboratories) at a constant current of 300 mA for 3 h at 4 °C. Equal loading and transfer were thereafter confirmed by staining the membranes with MemCode Reversible Protein Stain Kit (Thermo Scientific)^28^. For each set of target proteins, all samples from each subject were loaded to the same gel, which were all run at the same time.

After blocking for 1 h at room temperature in Tris-buffered saline (TBS; 20 mM Tris base, 137 mM NaCl, pH 7.6) containing 5% non-fat dry milk, the membranes were incubated overnight with commercially available primary antibodies diluted in TBS supplemented with 0.1% Tween-20 containing 2.5% non-fat dry milk (TBS-TM). They were then washed with TBS-TM and incubated for 1 h at room temperature with secondary antibodies conjugated with horseradish peroxidase; washed again with TBS-TM (2 × 1 min, 3 × 10 min) and then TBS (4 × 5 min) and, finally, the target proteins were visualized by application of the Super Signal West Femto Chemiluminescent Substrate (Thermo Scientific) followed by detection with the Molecular Imager ChemiDoc XRS system. The bands detected were quantified utilizing the contour tool in the Quantity One software, version 4.6.3 (Bio-Rad Laboratories). Prior to blocking, the membranes from each gel for each target protein were cut into strips and then assembled, so that all of the membranes with samples from any individual were exposed to the same blotting conditions. Images of the full-length membranes are displayed in Supplementary Fig. S2.

Following this visualization, the membranes were stripped of the phosphospecific antibodies using Restore Western Blot Stripping Buffer (Thermo Scientific) for 30 min at 37 °C, after which they were washed and re-probed with primary antibodies for the corresponding total protein in the same manner as described above. The levels of all phospho-proteins were normalised to the corresponding level of total protein. The levels of MuRF-1, MAFbx, REDD1 and rpS6 were normalized against the total protein staining obtained with the MemCode kit.

### Immunoprecipitation (IP)

To immunoprecipitate S6K1 and eIF4E, 3–4 mg muscle tissue was homogenized in ice-cold lysis buffer containing 40 mM Hepes (pH 7.5), 120 mM NaCl, 1 mM EDTA, 10 mM sodium pyrophosphate, 50 mM NaF, 0.5 mM Na_3_VO_4_, 10 mM β-glycerophosphate, 1% (v/v) Halt Protease Inhibitor Cocktail (Thermo Scientific) and 0.3% (w/v) CHAPS detergent. Next, these homogenates were rotated for 30 min and then centrifuged at 10,000×*g* for 10 min at 4 °C, after which the supernatant was collected and its protein concentration determined using the Pierce 660 nm protein assay (Thermo Scientific). The remaining pellet was stored at − 80 °C for later analysis.

In the case of S6K1, muscle lysates containing 750 µg protein were incubated with 7.2 µg rabbit anti-S6K1 antibody (sc #230, SantaCruz Biotechnology, Heidelberg, Germany) and 10 µl protein-A Sepharose beads (GE Healthcare, Uppsala, Sweden) overnight at 4 °C with rotation. For eIF4e, 500 µg protein was incubated with 5 µg mouse anti-eIF4E antibody (sc #271480) and 15 µl protein G magnetic beads (Thermo Scientific) for 4 h. Following these incubations, the beads with the bound immunocomplexes were spun down or trapped using a magnetic rack and washed twice in lysis buffer containing 0.5 M NaCl. For S6K1, the beads were subjected to a final wash in kinase-specific assay buffer prior to the kinase assays (see below). The magnetic beads with bound eIF4e were suspended in 1 × LSB 100 µM DTT, boiled for 10 min at 70 °C and then immunoblotted for eIF4E and 4E-BP1, as described above. This analysis was performed only on Pre- and Post-samples, due to a lack of muscle tissue, and, moreover, since 4E-BP1 phosphorylation was found to be altered only immediately after exercise.

### Kinase assay

The kinase assay was performed in accordance with the procedure described by McGlory et al.^29^. After washing in kinase-specific assay buffer (50 mM Tris pH 7.5, 0.03% BrijL23 and 0.1% βME), the beads were suspended in 60 µl assay buffer and this mixture then divided into three aliquots of 20 µl each. Two of these received 5 µl 300 µM synthetic S6K1 substrate (KRRRLASLR) and the third (the blank) 5 µl assay buffer without substrate. The assay was initiated by addition of 25 µl of a radioactive kinase-specific reaction mix, incubated for 60 min at 30 °C on a rotating platform and terminated with 50 µl phosphoric acid (1% v/v). The final concentrations in the reaction mix (50 µl) were 100 µM ATP, 10 mM MgCl_2_, ^32^γ-ATP (specific activity: ~ 3.0 × 10^6^ cpm ×  nmol^−1^) and 30 µM S6K1 substrate.

Subsequently, 75 µl of each sample was spotted onto p81 filter paper (GE Healthcare) and washed three times in phosphoric acid and once in acetone. When the filter paper had dried, it was immersed in scintillation fluid (FilterSafe, Zinsser Analytic GmbH, Frankfurt, Germany) and placed in a liquid scintillation counter (Beckman Coulter AB, Bromma, Sweden). The blank was subtracted from the average value of the duplicate assays and the values obtained expressed as pmol × min^−1^×mg^−1^ protein^29^.

### Antibodies

For immunoblotting, primary antibodies against Akt (Ser^473^, #9271; total, #9272), PRAS40 (Thr^246^, #2997; total, #2691), TSC2 (Thr^1387^, #5584; total, #3635), mTOR (Ser^2448^, #2971; total, #2983), S6K1 (Thr^389^, #9234; total #2708), rpS6 (Ser^235/236^ #2211), 4E-BP1 (Thr^37/46^, #2855; total, #9644), eIF4e (total, #9742), eEF2 (Thr^56^, #2331; total, #2332), AMPK (Thr^172^, #4188; total, #2532) and p38 (Thr^180/182^, #9211; total, #9212) were all purchased from Cell Signaling Technology (Beverly, MA, USA). Primary antibodies against total MAFbx (#92281) and REDD1 (#63059) were purchased from Abcam (Cambridge, UK) and total MuRF-1 (#sc-32920) antibody from Santa Cruz Biotechnology (Heidelberg, Germany). All primary antibodies were diluted 1:1000, except in the case of phospho-eEF2, where the dilution was 1:2000. Secondary anti-rabbit antibodies (#7074; 1:10,000) were purchased from Cell Signaling Technology and secondary anti-goat antibodies (#ab7132; 1:10,000) from Abcam.

### RNA extraction and quantitative real-time PCR (qRT-PCR)

The procedure employed for quantification of mRNA has been described in detail previously^30^. In brief and with minor modifications, approx. 3 mg lyophilized muscle was homogenized in PureZOL RNA isolation reagent (Bio-Rad Laboratories) using the BulletBlender and RNAse-free 0.5-mm ZrO_2_ beads, the total RNA extracted and its purity confirmed (260/280 nm ratio of 1.90), and 2 μg converted into cDNA. Subsequently, 25 µl qRT-PCR amplification mixtures containing the template cDNA in RNase-free water, 2 × SYBR Green Supermix (Bio-Rad Laboratories) and appropriate primers were prepared for thermal cycling on a Bio-Rad iCycler (Bio-Rad Laboratories). The relative changes in mRNA levels were analysed with the 2^−ΔCT^ procedure, using GAPDH mRNA as reference, which was stable across time and condition. The concentration of cDNA, annealing temperature and PCR cycle protocol were optimized for each primer pair.

### RNA sequencing and data analysis

To gain a deeper understanding of the interactions between endurance and resistance exercise performed with different muscles, we performed an exploratory global analysis of gene expression by RNA-Seq on triceps biopsies from three subjects (according to the cross-over design two subjects performed ER-Arm as first trial and one subject began with R-Arm), at the following time points: prior to exercise, immediately after (time 0) and 90 min after exercise (time 90) to account for early changes in the transcriptome. Muscle tissue for these analyses was only available from three subjects due to the previous extensive analysis on samples from a small muscle group.

Freeze dried muscles were pulverized using a dry ice-cold mortar and pestle. Samples were afterwards homogenized in TriReagent lysis reagent (Sigma, MO, USA) with metal beads using a TissueLyzer II (Quiagen, Hilden, Germany). RNA was purified according to the manufacturer’s instructions and cleaned using Nucleospin II RNA columns (Macherey–Nagel, Düren, Germany). Quality of RNA was determined using chip-based Bioanalyzer (Agilent, CA, USA) and 1 ug was used for RNA sequencing. RNA sequencing was completed by Eurofins Genomics (Ebersberg, Germany). Libraries were prepared using the Illumina TruSeq Stranded mRNA Library Preparation Kit. The pool was loaded onto Illumina HiSeq 2500 High Output flow cell and sequenced in a 1 × 50 bp single read format. Base calling was done by Illumina Real Time Analysis (RTA) v1.18.64 and output of RTA was demultiplexed and converted to FastQ format with Illumina Bcl2fastq.

Quality control of raw reads was determined using FastQC tool kit (Babraham Bioinformatics, http://bioinformatics.babraham.ac.uk/projects/fastqc). The reads were then aligned with reference genome of *Homo sapiens* (GRCh38.p13) downloaded from NCBI using STAR aligner tool^31^ and reads aligning to gene exons were counted using Featurecounts program^32^. List of differentially expressed genes (DEGs) between individual training protocols immediately after and 90 min after exercise was obtained by analyzing raw counts using DESeq2 package^33^ and using ashr package for log fold-change shrinkage^34^. Considering the study design, all genes showing p-value (adjusted by Benjamini–Hochberg method) < 0.05 were further examined for functional processes and differential expression. Panther classification system (http://www.pantherdb.org/) was used for gene ontology analysis of biological processes and functional annotation of differentially expressed genes. For clustering analysis, gene expression was normalized and kmeans clustering was performed using Hartigan–Wong algorithm. We determined the optimal number of clusters using elbow method on total within-cluster sum of squares. Genes within clusters were ranked based on the fold-change magnitude immediately after and 90 min after exercise normalized by total standard deviation of all data points. Genes were then analyzed by GSEA^35^, using gene curated gene set of canonical pathways (MsigDB.C2.CP). Venn diagram visualization of pathways common between individual clusters was performed using UpSetR package^36^.

### Statistical analyses

Parametric statistical analyses were employed, and all values are presented as means ± standard deviation (SD). A two-way repeated measures ANOVA (time and trial) was used to evaluate changes in intracellular signalling, kinase activity, FSR, the levels of muscle glycogen and lactate, and plasma concentrations. Fisher’s LSD post-hoc test was performed if main effects (P < 0.05) or interaction effects (P < 0.10) were present. A P-value < 0.05 in the post-hoc analysis was considered statistically significant. Unless otherwise stated, all P-values presented hereafter were obtained in the post-hoc analysis. All statistical analyses were performed using the STATISTICA software version 13.0 (Dell Software, CA, USA) and all the data supporting the findings of this study are available from the corresponding author upon reasonable request.

## Results

### Exercise parameters

All subjects completed both trials in accordance with the criteria chosen and performed the same amount of work during resistance exercise in both trials. The total number of repetitions was 144 ± 3 in both trials and the total time under tension 416 ± 47 and 406 ± 32 s for the R-Arm and ER-Arm trials (*P* > 0.05), respectively. The average work rate during interval cycling was 258 ± 31 W, corresponding to 83 ± 3% of individual VO_2peak_.

### Plasma parameters

**Figure 2:**
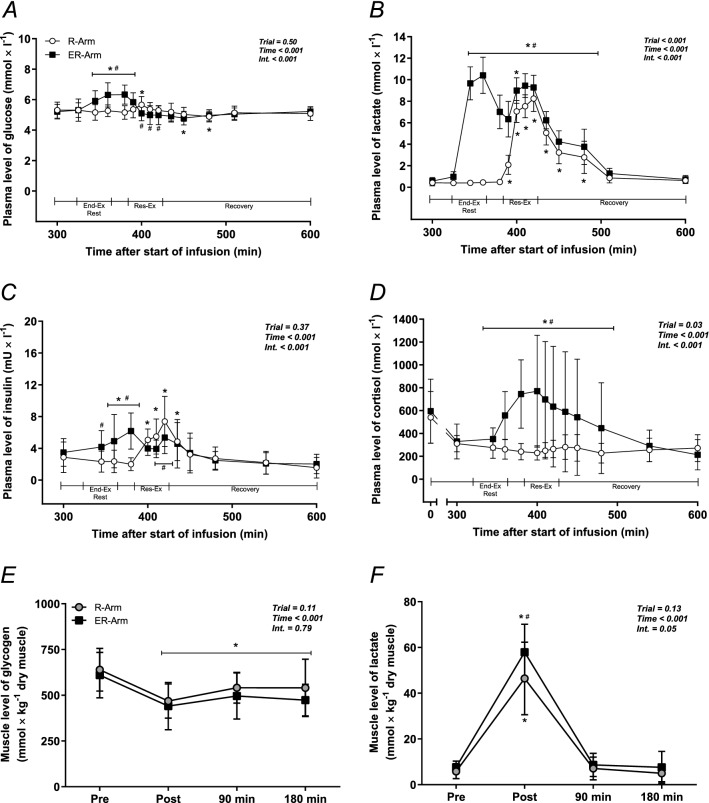
Plasma levels of (**A**) glucose, (**B**) lactate, (**C**) insulin and (**D**) cortisol at rest, during exercise and recovery, as well as muscle levels of glycogen (**E**) and lactate (**F**) at rest and after exercise in the two trials. The values in these graphs are means ± SD for 8 subjects (n = 7 for muscle lactate). *P < 0.05 vs. Rest, ^#^*P* < 0.05 vs. the R-Arm trial.

During interval cycling (the ER-Arm trial) plasma glucose levels rose from 5.2 ± 0.5 to 6.3 ± 0.8 mmol l^−1^ and remained elevated until the warm-up prior to the resistance exercise (*P* < 0.05). During the resistance exercise, plasma glucose fell to levels significantly lower than those in the R-Arm trial (Fig. 2A).

In connection with the ER-Arm trial, plasma levels of lactate increased significantly during cycling, reaching a concentration of 10.4 ± 1.7 mmol l^−1^ immediately after exercise (*P* < 0.05, Fig. 2B) and remaining elevated above both baseline and those in the R-Arm trial until after 90 min of recovery from resistance exercise. In the case of the R-Arm trial, lactate levels peaked immediately after the last set of resistance exercise at 8.2 ± 1.2 mmol l^−1^ and remained higher than at rest for 90 min of recovery (*P* < 0.05).

The fasting levels of insulin were 3 mU l^−1^ in both trials and did not change in response to isotope infusion. These levels were increased with both exercise protocols, although the average concentration did not exceed 8 mU l^−1^ at any time-point during the two trials (Fig. 2C).

Plasma levels of cortisol fell significantly from 540 ± 227 to 311 ± 72 and from 595 ± 280 to 310 ± 126 nmol l^−1^ during the five hours of rest (time: 0 – 300 min) in the R-Arm and ER-Arm trials, respectively. Interval cycling increased the cortisol level significantly to 786 ± 482 nmol l^−1^ in the ER-Arm trial (*P* < 0.05, Fig. 2D, time: 400 min). These levels then remained 46% higher than at rest for 60 min (time: 480 min) after the resistance exercise (*P* < 0.05). During the R-Arm trial, plasma levels of cortisol were unaltered by either the exercise or recovery.

With the exception of cortisol, plasma glucose, lactate and insulin did not change significantly during the five hours (0–300 min) of rest with isotope infusion prior to the exercise protocol (data not shown).

### Muscle glycogen and lactate

The level of muscle glycogen declined by ~ 28% (*P* < 0.05) during exercise in connection with both the R-Arm and ER-Arm trials. During recovery, glycogen was resynthesized in both cases, resulting in levels 16% (R-Arm trial) and 7% (ER-Arm trial) higher than immediately after exercise (*P* < 0.05) with no difference between trials (Fig. 2E).

Muscle concentrations of lactate increased significantly during exercise in both trials, although to a greater extent in the ER-Arm trial compared to the R-Arm trial (*P* < 0.05, peak value of 58.0 ± 12.3 vs. 46.4 ± 15.8 mmol kg^−1^ dry muscle, respectively, n = 7). During recovery these levels returned to baseline in both cases (Fig. 2F).

### Isotope enrichment and the FSR of mixed muscle protein

**Figure 3:**
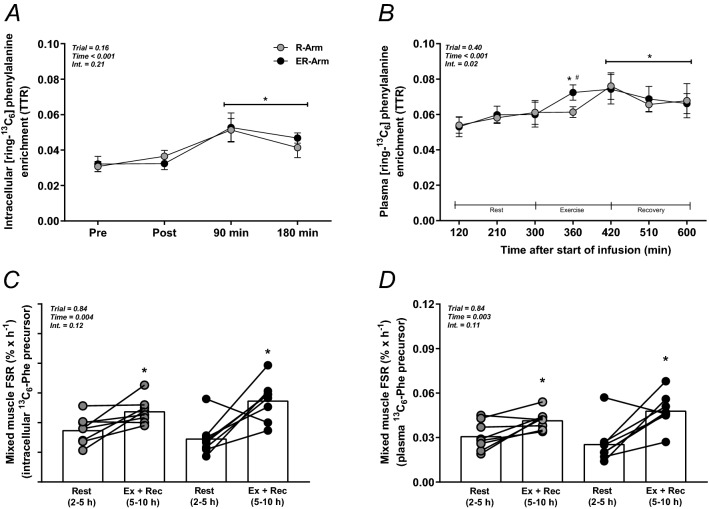
The FSR of mixed muscle protein based on intracellular (**A**, **C**) and plasma (B, **D**) enrichment of L-[ring-13C6]-phenylalanine (tracer to tracee ratio: TTR) in both trials. Individual and mean values of FSR (**C**, **D**) over 300 min of exercise and recovery. *P < 0.05 vs. Rest, ^#^*P* < 0.05 vs. the R-Arm trial.

The mean intracellular enrichments (tracer to tracee ratio; TTR) at rest were 0.031 ± 0.003 and 0.032 ± 0.005 in the R-Arm and ER-Arm trials, respectively. Immediately following exercise the intracellular TTR rose to 0.052 ± 0.006 and 0.053 ± 0.008 (both *P* < 0.05), respectively, falling again during recovery in both cases 0.044 ± 0.004 (*P* < 0.05) (Fig. 3A). At rest, the mean plasma enrichments were 0.054 ± 0.006 and 0.053 ± 0.003 in the R-Arm and ER-Arm trials, respectively. Following cycling exercise in the ER-Arm trial and resistance exercise in the R-Arm corresponding values increased by approximately 40% in both trials (*P* < 0.05), remaining significantly higher than at rest throughout the trial (Fig. 3B).

The FSR of mixed muscle protein at rest, calculated using the intracellular enrichment as the precursor pool, was 0.053 ± 0.014% h^−1^ in the R-Arm trial and 0.044 ± 0.018% h^−1^ in the ER-Arm trial, with slower rate of synthesis in 6 of our 8 subjects in the latter case. Subsequently, FSR calculated for the entire 300 min, i.e., for both the exercise and recovery periods, the rates were 0.072 ± 0.005% h^−1^ (1.36-fold increase) and 0.083 ± 0.007% h^−1^ (1.89-fold increase) in the R-Arm and ER-Arm trials, respectively. ANOVA revealed a main effect of time and elevation above rest in both cases (*P* < 0.05 vs. Rest, Fig. 3C).

Using plasma enrichment as the precursor pool for calculation, the FSR at rest in the R-Arm and ER-Arm trials was 0.031 ± 0.010 and 0.026 ± 0.013% h^−1^, respectively, increasing during the subsequent 300 min of exercise and recovery to 0.042 ± 0.006 (1.35-fold increase) and 0.048 ± 0.012% h^−1^ (1.85-fold increase, both *P* < 0.05 vs. Rest, Fig. 3D), with no difference between trials.

### Intracellular signalling and kinase activity

The degree of phosphorylation of Akt^Ser473^ did not change at any time-point during either of the trials. However, phosphorylation of PRAS40^Thr246^, a downstream target, decreased by 38% and 53% (both *P* < 0.001) after exercise in the R-Arm and ER-Arm trials respectively, but this reduction was reversed during recovery (Supplementary Fig. S1).

**Figure 4:**
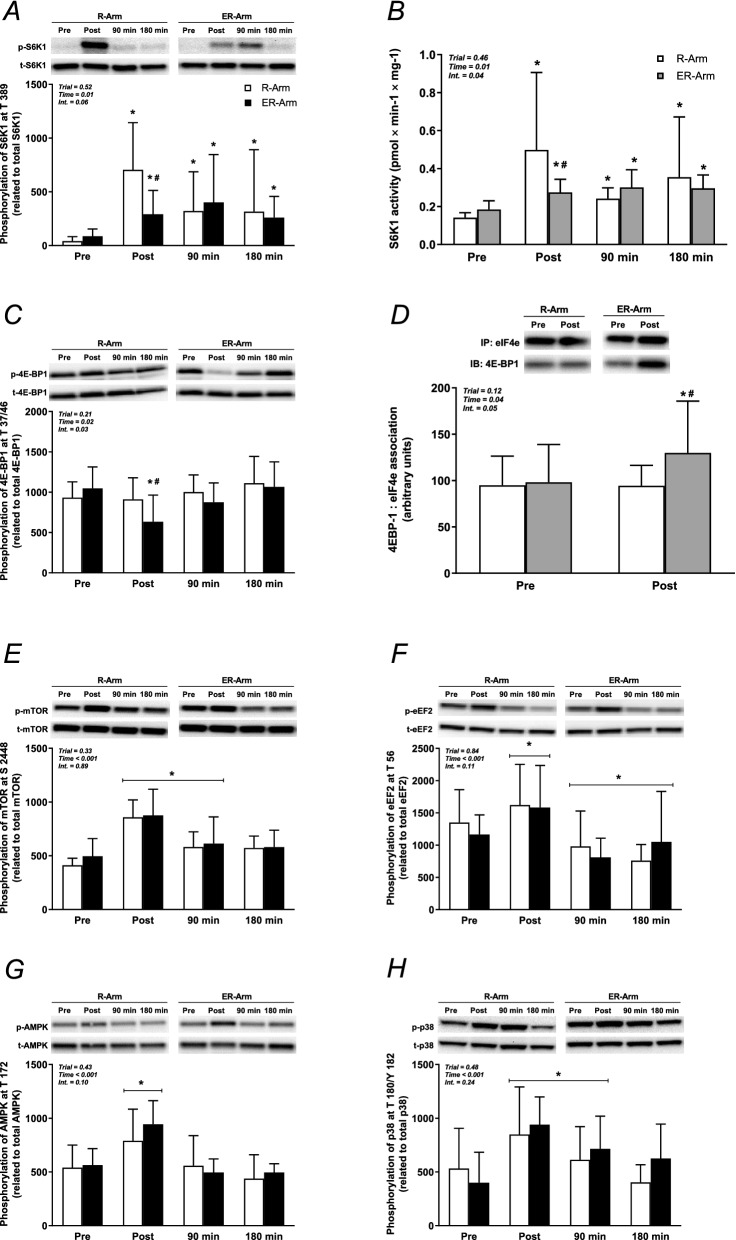
Phosphorylation of S6K1 at Thr^389^ (**A**), kinase activity of S6K1 (**B**), phosphorylation of 4E-BP1 at Thr^37/46^ (**C**), the total amount of 4E-BP1 immuoprecipitated together with eIF4E (**D**), phosphorylation of mTOR at Ser^2448^ (**E**), eEF2 at Thr^56^ (**F**), AMPK at Thr^172^ (**G**) and p38 at Thr^180^/Tyr^182^ (**H**) at different stages of both trials. The values presented are means ± SD for 8 subjects. *P < 0.05 vs. Rest, ^#^*P* < 0.05 vs. the R-Arm trial. Symbols denoted with a line represent a main effect. Symbols without a line represent an interaction effect with corresponding significant differences in the post-hoc analysis. Representative immunoblots (from strips of full-length blots) of both phosphorylated (upper panel, except for IP) and total protein (lower panel) from one subject are shown above each graph. The two sets of bands have been rearranged, and separated with a white space, to fit the order of the trials in the graphs. Pre = baseline, Post = immediately after exercise, 90 and 180 min = length of recovery.

In both trials, S6K1^Thr389^ phosphorylation was elevated immediately after exercise, more so in the R-Arm trial (11-fold versus fivefold in the ER-Arm trial, *P* < 0.05). In the biopsies taken 90- and 180-min post-exercise the extent of phosphorylation at Thr389 was similar between trials, but still 4 to sixfold greater than at rest (*P* < 0.05) (Fig. 4A). The mean activity of S6K1 at rest was 0.163 ± 0.36 pmol min^−1^ mg protein^−1^, similar in both trials, increasing immediately following exercise by 69% (*P* < 0.05) in the ER-Arm trial, and significantly more (205%, P < 0.05) in the R-Arm trial. After 90 and 180 min of recovery this activity remained elevated, but to the same extent in both trials (Fig. 4B). The S6K1 activity agreed well with the degree of S6K1^Thr389^ phosphorylation (r = 0.77, P < 0.01). Phosphorylation of S6 at Ser^235/236^ was higher immediately after exercise than at rest in both the R-Arm and ER-Arm trials (10- and 9-fold, respectively, *P* < 0.05 in both cases). Following 90 min of recovery, these levels remained elevated but had returned to baseline after 180 min (Supplementary Fig, S1).

In the case of 4EBP-1^Thr37/46^, another downstream target of mTOR, phosphorylation was reduced by 36% immediately after exercise in the ER-Arm trial (*P* < 0.05 vs. Rest and *P* < 0.05 vs. R-Arm at that time point), but unaltered in the case of the R-Arm trial (Fig. 4C). 4EBP-1^Thr37/46^ phosphorylation did not differ significantly between trials or from baseline after 90 and 180 min of recovery. The interaction between eIF4e and 4E-BP1 increased 33% (*P* < 0.05 vs. Rest and *P* < 0.05 vs. R-Arm at that time point) following exercise in the ER-Arm trial, with no change in the R-Arm trial (Fig. 4D).

Phosphorylation of mTOR at Ser^2448^ was significantly elevated approximately 90%, immediately after exercise during both trials. During recovery, this phosphorylation fell somewhat but remained elevated after 90 min in both trials (*P* < 0.05 Fig. 4E). The phosphorylation of eEF2^Thr56^ was increased 26–29% immediately following exercise in both trials (*P* < 0.05), but fell again at mid- and late recovery to a level 10–44% lower than at baseline (*P* < 0.05), with no differences between trials (Fig. 4F).

Immediately following exercise, the extent of AMPK^Thr172^ phosphorylation was enhanced 43% and 71% in the R-Arm and ER-Arm trials, respectively (*P* < 0.05 vs. Rest). At 90- and 180-min post-exercise these levels had returned to baseline, with no difference between trials (Fig. 4G). TSC2, the downstream target of AMPK, demonstrated more phosphorylation at serine 1387 immediately post-exercise (approximately 25% in both trials; *P* < 0.05), an increase maintained after 90 min of recovery, but reversed after 180 min (Supplementary Fig. S1). Phosphorylation at the Thr 1462 residue of this protein was not affected by either protocol. The level of p38 MAPK^Thr180/182^ was increased 82% and 101% immediately following exercise in the R-Arm and ER-Arm trial, respectively, (*P* < 0.05 in both cases), with no significant difference between them. This elevation was still present after 90, but not 180 min of recovery (Fig. 4H).

### mRNA and protein levels

**Figure 5:**
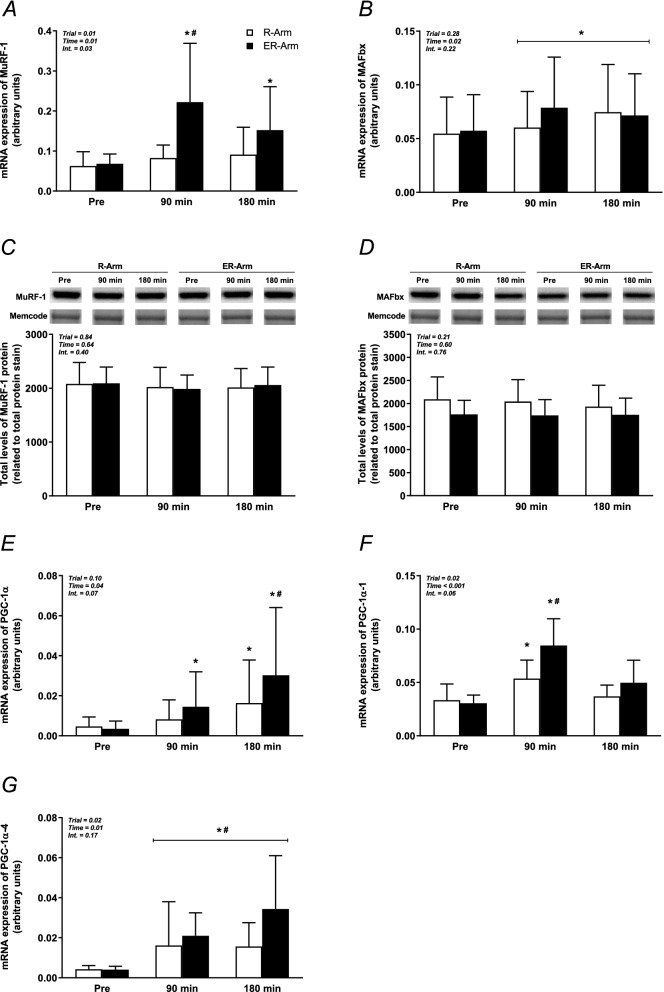
The levels of MuRF-1 (**A**, **C**) and MAFbx (**B**, **D**) mRNA and protein, as well as of PGC-1α1 (**E**), PGC-1α4 (**F**) and PGC-1α1 (**G**) mRNA in muscle biopsies taken at rest and following 90 and 180 min of recovery during both trials. The values presented are means ± SD for 8 subjects. *P < 0.05 vs. Rest, ^#^*P* < 0.05 vs. the R-Arm trial. Symbols denoted with a line represent a main effect. Symbols without a line represent an interaction effect with corresponding significant differences in the post-hoc analysis. The mRNA levels were normalized to that of GAPDH mRNA and analyzed using the 2^−ΔCT^ procedure. The level of protein was related to the Memcode™ protein stain, with representative bands (from strips of full-length blots) from one subject shown above each graph. The bands have been rearranged, and separated with a white space, to fit the order of the trials in the graphs.

In the ER-Arm trial, the level of MuRF-1 mRNA was elevated 2 to threefold after 90 and 180 min of recovery (*P* < 0.05 vs. Rest and *P* < 0.05 vs. R-Arm at both time points), with no such change in the case of the R-Arm trial (Fig. 5A). The levels of MAFbx mRNA was increased 20–45% during recovery, with no difference between the trials (*P* = 0.02; Fig. 5B). MuRF-1 and MAFbx proteins levels did not change significantly during either trials (Fig. 5C,D), as was also the case for the level of total REDD1 protein (Supplementary Fig. S1).

In the ER-Arm trial, following 90 and 180 min of recovery, the level of PGC-1α mRNA was increased 4.2- and 8.7-fold (*P* < 0.05 vs. Rest and *P* < 0.05 vs. R-Arm for the latter time point), respectively, but only 3.5-fold at 180 min in the R-Arm trial (*P* < 0.05 for time, Fig. 5E). The level of the PGC1-α1 isoform (exon 1a) were 62 and 178% higher than at baseline following 90 min of recovery in the R-Arm and ER-Arm trials, respectively, with the latter increase being significantly greater (*P* < 0.05 vs. Rest and *P* < 0.05 vs. R-Arm at that time point, Fig. 5F). The level of the PGC1-α4 isoform (exon 1b, truncated) was elevated 3.8-fold at 90 min of recovery in the R-Arm trial and remained so 180 min after exercise. In the ER-Arm trial, corresponding level was increased 5.2- and 8.5-fold at 90 and 180 min of recovery, respectively, (*P* < 0.05 for time and for trial, Fig. 5G).

### RNA-seq analysis

**Figure 6:**
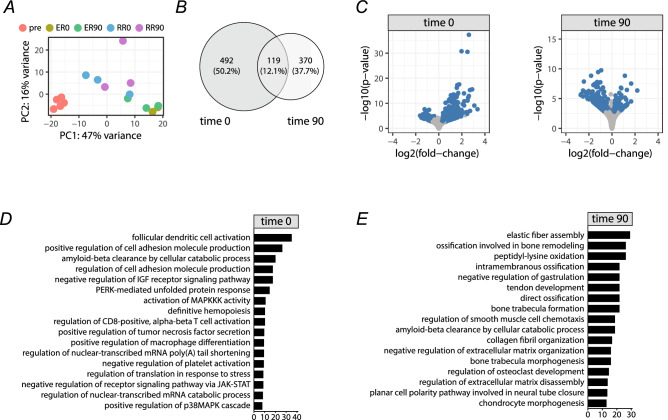
Initial analysis of ER-Arm: R-Arm RNASeq data. PCA plot of individual samples shows good clustering of pre-training samples and individual exercise protocols (**A**). Analysis of differentially expressed genes identified 611 genes expressed differentially at time 0 min (immediately after the exercise) and 489 genes expressed differentially 90 min after the exercise. Out of these, 119 were common (**B**). Volcano plots of expression fold changes with relation to p-values for individual analyzed time points. R-Arm protocol was chosen as a baseline (**C**). Top PantherDB-Gene Ontology pathways identified based on differential expression of genes between ER-Arm and R-Arm exercise protocols at two different time points (0 min, 90 min) (**D**, **E**).

PCA analysis of gene expression data from ER-Arm and R-Arm protocols prior to, immediately post-exercise and 90 min into recovery displayed good clustering of individual samples, with biggest differences between protocols and smaller differences between time points within each protocol (Fig. 6A). Initial analysis showed 611 differentially expressed genes between trials immediately post-exercise (time 0 min) and 489 genes 90 min post-exercise (time 90) with a small overlap of 119 genes that are common to both time points (Fig. 6B,C). Gene ontology analysis using Panther classification tool on differentially expressed genes identified several significantly overrepresented pathways at both time points. Out of these, it is interesting to mention that immediately post-exercise (time 0) the main difference between protocols seems to be in the ability to activate pathways connected to immune system, MAPK mediated stress response, hematopoiesis, transcription, translation and cell adhesion (Fig. 6D). On the other hand, the pathway signature at 90 min post-exercise (time 90) seems to shift towards bone, muscle and extracellular matrix remodeling, assembly of elastic and collagen fibers, and signaling pathways connected to cell migration and morphogenesis (Fig. 6E).

### Gene expression clustering

In order to understand the underlying mechanisms by which ER-Arm or R-Arm protocols might influence the physiological response and pinpoint the contributions of aforementioned pathways, we used k-means clustering to find genes with concordant expression profiles.

**Figure 7:**
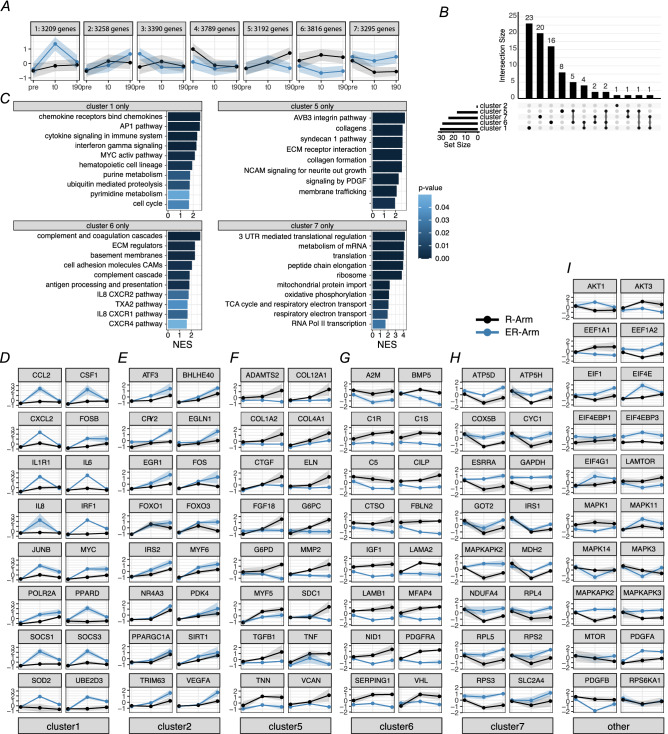
Clustering of gene expression for R-Arm (black lines) and ER-Arm (blue lines) RNASeq at different time points (0 min, 90 min). K-means clustering based on gene expression and fold-changed in individual time points identified 7 cluster with distinct expression profiles (**A**). Pathway analysis of individual clusters by GSEA, with genes ranked by fold-change/standard deviation ratio identified both distinct and common pathways, shown as upset-plot (**B**). Pathways exclusive to individual clusters with their respective normalized enrichment score (NES) are plotted with their p-value indicated by color (**C**). Only single pathway (Smad2/3 nuclear signaling) has been identified for cluster 2 and is not shown. Expression profiles of selected genes for individual clusters (**D**–**H**). Expression profiles of selected genes that either play role in protein synthesis or show interesting complementary expression patterns (**I**).

Clustering of the genes based on their normalized expressions revealed that they roughly fall into 7 clusters (Fig. 7A). Out of these, cluster 3 and 4 show different gene expression in samples taken before training and were therefore excluded from further analysis. The remaining clusters fall into the following profiles: (i) Genes whose expression changed rapidly right after the end of exercise but normalized at 90 min. It should be noted that only the in ER-Arm protocol shows cluster with this pattern (cluster 1) (ii) Genes whose expression did not markedly differ immediately post-exercise (time 0), but started to diverge at 90 min post-exercise in either ER-Arm or R-Arm protocols (cluster 2 and 5, respectively). (iii) Genes whose expression diverged immediately post-exercise and where the difference persisted (clusters 7 and 6, respectively). These 5 clusters are of interest, since they might account for some of the physiological changes and explain the reinforcement or interference of concurrent training protocols.

In order to gain insight into the possible physiological role of different gene clusters, we performed a Gene Set Enrichment Analysis on genes ranked by their difference in expression normalized to their respective standard deviations. We focused on finding canonical pathways that are part of molecular signature database (MsigDB.C2.CP). There is a minor overlap in enriched pathways in between the clusters, but the majority of them belong to a single cluster (Fig. 7B). Clusters 1, 5, 6, 7 have 23, 20, 16, and 8 unique identified pathways, respectively.

Analyzing the pathways in per-cluster fashion assigns some of the previously identified ones to a particular expression profile. Immune response and cytokine signaling as well as early response transcription factors and unfolded protein response are overrepresented in cluster 1. Transcription, translation and changes in cellular respiration are overrepresented in cluster 7 and both are favored by ER-Arm protocol. On the other hand, cell adhesion, extracellular matrix remodeling and assembly of collagen and elastic fibers are favored by RR-Arm protocol (clusters 5 and 6) (Fig. 7C).

When we inspected expression profiles of selected genes of interest, we could see many early response transcription factors (*JUNB*, *MYC*, *FOS PPARD*), inflammation-related genes (*IRF1*, *IL6*, *CCL2*, *CXCL2*, *SOCS1*, *SOCS3*) as well as genes related to oxidative stress (*SOD2*) and proteolysis (*UBE2D3*) in cluster 1 (Fig. 7D). Analysis of cluster 2 identified many genes that are canonically connected to exercise responses such as *TRIM63 (MuRF-1)*, *PPARGC1A* (PGC-1α, *VEGFA*, *ATF3*, *FOXO1*, *FOXO3*, *NR4A3* together with genes that influence response to hypoxia (*EGLN1*, *BHLHE40*) and *MYF6* that can influence myogenic programs in response to injury (Fig. 7E). Cluster 7 contains genes that are upregulated early in ER-Arm protocol and the difference persists in time. Genes influencing oxidative metabolism (*ATP5D, ATP5H*, *COX5B*, *CYC1*, *ESSRA*, *NDUFA4*), fuel utilization (*SLC4A2*, *GOT2*, *MDH2*, *GAPDH*), and translation (*RPL4*, *RPL5*, *RPS2*, *RPS3*) are particularly enriched (Fig. 7H).

On the other hand, clusters 5 and 6 contain genes that are upregulated either immediately or later after the end of the exercise bout in the R-Arm protocol. Cluster 5 (Fig. 7F) contains many genes related to elastic and collagen fiber assembly (*COL12A1*, *COL1A2*, *COL4A1*, *ELN*), extracellular matrix remodeling (*ADAMTS2*, *CTGF*, *VCAN*, *MMP2*) and muscle size and development (*FGF18*, *MYF5*, *SDC1*, *TGFB1*, *TNF*, *TTN*). Finally, cluster 6 with early divergence in expression and upregulation in R-Arm cluster does not have a particularly strong signature of specificity in expression, but contains several interesting genes, including complement cascade (*C1R*, *C1S*, *C5*, *SERPING1*), extracellular matrix (*LAMA2*, *LAMB*, *1FBLN2*), hypoxia (*PDGFRA*, *VHL*) and *IGF1* (Fig. 7G). Genes of interest, independent of cluster, are displayed in Fig. 7I.

## Discussion

Recently, there has been an increasing interest in the molecular mechanisms underlying muscle adaptation to various combinations of endurance and resistance exercise, in most cases involving the same muscle group. In this context, our protocol of interval leg cycling in combination with upper-body resistance exercise, has not been employed previously. The major findings were that preceding high-intensity interval exercise potentiated certain effects of resistance exercise on the triceps muscle, i.e., the elevations in PGC-1α1, PGC-1α4 and, in particular, MuRF-1 mRNA. Exploratory RNA sequencing, in a small subgroup of subjects, also revealed that 862 genes were differently expressed with ER-Arm during recovery compared with R-Arm. Furthermore, high-intensity interval cycling transiently attenuated the increases in S6K1 activity induced by resistance exercise and promoted interaction between 4E-BP1 and eIF4E immediately after the session of resistance exercise. This acute attenuation, which was no longer present following 90 and 180 min of recovery, was not related to any difference in the FSR of mixed muscle protein, which increased to a similar extent in both trials.

Exercising with the triceps muscle raised the levels of PGC-1α1 and, in particular, PGC-1α4 mRNA (Fig. 5E–G), with somewhat different time courses. Both isoforms were elevated after 90 min of recovery, but only PGC-1α4 remained elevated 180 min following exercise. This pattern resembles the changes we previously observed in the vastus lateralis muscle following heavy resistance exercise^12^. Interestingly, when high-intensity cycling was performed prior to the arm exercise, the increases in both PGC-1α1 and PGC-1α4 were considerably larger, which is in line with the reported increase in total PGC-1α following concurrent exercise with the same muscle^21,37,38^, but here noted for separate muscles, suggesting a systemic effect (Fig. 5E–G). The levels of p38 MAPK and CaMK, stimulators of PGC-1α1 proposed previously^39^, did not differ between conditions, whereas phosphorylation of AMPK tended to be higher during the ER-Arm trial, but to an extent too small to provide a probable explanation for rises in PGC-1α1 and PGC-1α4. Regardless of upstream mechanism, these results suggest that repeatedly performing the exercise protocol could enhance the oxidative capacity of the triceps muscle^8^.

Several exercise-induced alterations in systemic factors, such as catecholamines and IL-6 (via AMPK), appears to induce PGC-1α in experimental animals^40,41^. However, acute β-adrenergic stimulation did not affect the level of PGC-1α in human muscle, neither at rest nor following exercise^42,43^. Another potential candidate is lactate, which has been implicated as mediating direct or indirect signaling in various tissues, including skeletal muscle^44–46^. For instance, the Brook’s laboratory^47^ has shown that 10 mM lactate significantly increased the level of PGC-1α in L6 myoblasts cells. Importantly, the majority of these studies have evaluated PGC-1α gene expression as a single entity, most likely reflecting the combined expression of all coactivator variants. Indeed, studies evaluating the regulation of PGC-1α isoform-specific expression by the α-adrenergic agonist clenbuterol reported an elevation in total PGC-1α expression, concomitant with a reduction in PGC-1α1 and an increase in PGC-1α4 levels^48^.

In the present study, muscle levels of lactate were 25% higher after resistance exercise in the ER-Arm than the R-Arm trial (46.4 vs. 58.0 mmol kg^−1^ dry muscle or 10.8 vs. 13.5 mmol kg^−1^ wet muscle). This was probably due to an increased uptake of leg muscle produced lactate^49,50^, together with less efflux from the muscle due to a smaller muscle-to-blood gradient of lactate in the former trial^51^. However, accumulation of lactate due to activation of the triceps muscle during the cycling exercise may also have contributed to the higher levels. We found no relationship between muscle or plasma levels of lactate and changes in mRNA encoding the two isoforms of PGC-1α in the triceps muscle. Similarly, cycling at various intensities produced different amounts of lactate, but similar increases in the level of PGC-1α mRNA in the vastus lateralis muscle^43^. Thus, in contrast to the observations on incubated muscle cells, studies on human muscle provide no support for a role of lactate in stimulating PGC-1α in human muscle.

The MuRF-1 and MAFbx genes are readily induced in a number of animal models and their products play pivotal roles in proteolysis^52^. Most previous investigations found that acute bouts of resistance or endurance exercise induce the expression of MuRF-1, while reducing or not altering that of MAFbx^30,53–55^. Here, resistance arm exercise did not change the levels of MuRF-1 mRNA, whereas that of MAFbx rose slightly. However, when interval cycling preceded this exercise, the level of MuRF-1 mRNA was markedly increased, again suggesting a systemic effect (Fig. 5A). Similarly, we and others have reported that an acute bout of concurrent exercise involving the quadriceps muscles increased the level of MuRF-1 mRNA more than resistance exercise alone^20,37^. This increased proteolytic gene expression must however not be interpreted as a diminished hypertrophy response, rather a response that facilitates muscle remodeling^56^.

This increase in MuRF-1 mRNA could have been due to changes in AMPK activation, but it is questionable whether the relatively minor differences in AMPK phosphorylation (20%) here contributed to the major alteration in the level of this mRNA. Furthermore, phosphorylation of p38 and Akt was unaltered. The MuRF-1 promoter contains both FOXO-binding and glucocorticoid receptor (GR)-binding elements, with activation of GR alone promoting transcription^57^. Therefore, the 3.4-fold higher cortisol levels during ER-Arm than the R-Arm trial may possibly have stimulated GR and thereby increased the level of MuRF-1 mRNA during recovery. It should, however, be emphasized that the peak cortisol level during the ER-Arm trial (at approximately 11:00 AM) was within the normal diurnal range and only 30% higher than at baseline (6:00 AM). In our previous investigation the increased level MuRF-1 mRNA was associated with more of the corresponding protein, but this was not the case here. Altogether, our present data indicate that exercise can elevate MuRF-1 not only via intrinsic, but also systemic factors.

On the basis of earlier observations^4,22,23^, we hypothesized that endurance exercise would potentiate the stimulation of mTORC1 signaling in the triceps by resistance exercise. In contrast, the lower S6K1 activity and more extensive interaction between 4E-BP1 and eIF4E immediately after exercise in the ER-Arm trial indicate acute interference with translation initiation. An acute and transient inhibition of 4E-BP1 in the *vatus lateralis* after cycling exercise has been illustrated previously^5,20^, but our data suggest that this inhibitory effect evoked by endurance exercise in one muscle also could be transferred to a remote contracted muscle. A finding that is surprising since an interference of anabolic signaling following concurrent exercise, using the same muscle, never has been reported in humans^20,37^. All other mTOR signaling proteins assessed, including rpS6, responded in a similar fashion to both trials. The extent of rpS6 phosphorylation did not reflect the level of S6K1 activity, which was unexpected, since the latter kinase activity is directed towards a peptide component of rpS6, at least in vitro. However, rpS6 can also be phosphorylated by Ras/ERK/RSK signaling^58^ and protein kinase A^59^, which may explain this discrepancy.

To elucidate the potential immediate effects of our different exercise protocols on protein synthesis, the FSR was calculated during the entire 300 min from the start of exercise to the completion of recovery. There were no differences between trials, with FSR increasing ~ 60% above rest (Fig. 3C,D). Although there was a numerically greater fold-change from rest in ER-Arm, this seemed to some extent to be explained by the non-significantly lower resting FSR in this trial, however, the FSR during exercise and recovery was in fact higher in 5 of the 8 subjects in the ER-Arm trial. The reason for the difference in resting FSR is not obvious but appear to be a natural variation^60^. This however indicates that the less pronounced increase in S6K1 activation immediately after exercise in the ER-Arm trial was not associated with a detectable difference in FSR. In this context mTORC1 signaling was enhanced to the same extent at 90 and 180 min post-exercise in both trials, so any potential effect of the difference immediately after exercise was probably short-lived. In support of this proposal, the reduction in 4E-BP1 phosphorylation after exercise was normalized after 15 min of recovery^5^. Thus, a small and short-lived reduction in translation does probably not significantly affect the incorporation of amino acids into proteins over a five-hour period, when also considering the methodological variability^61^. Here it must also be recognized that the signaling data provides a snapshot insight to the molecular changes, while FSR provides a more dynamic outcome, which highlights the necessity of time course approaches when assessing protein signaling. In agreement with previous observations in our own laboratory and others, free tracer enrichment was significantly increased after exercise^20,62,63^, by ~ 30% in the plasma and ~ 50% in muscle. The large increase and perturbations in intracellular enrichment less than one minute after the cessation of very intense exercise lead us to disqualify inclusion of FSR calculations over 180 min of recovery where the immediate post exercise biopsies intracellular enrichment is used as a precursor. As a note, this 180 min FSR calculation however, resulted in a tendency for increase above rest (*P* = 0.09), with no differences between trials. Altogether, all FSR calculations indicated increases of approximately 45–65% above rest, which is in line with previous investigations on exercise in the fasted state^64^.

Our targeted qRT-PCR analyses exhibited the most marked differences between protocols with regard to the induction of PGC-1α1, PGC-1α4 and MuRF-1 mRNA, which were more pronounced in the ER-Arm trial. We thus took an exploratory global transcriptomic approach in a subgroup of subjects to complement the qPCR analysis and gain more indicative insights into overall molecular changes. Despite the subgroup limitations, it was immediately notable that the preceding leg interval exercise induced markedly different transcriptional changes in the arm muscle. The enriched pathways we identified showed little overlap and the effect of time and trial seemed to be well defined. Clustering of the gene expression revealed five distinct gene profiles of interest. From those, we observed the immediate effect of interval endurance training on immune-related genes and early response transcription factors right after cessation of the entire exercise session (cluster 1). The genes in cluster 1 clearly illustrated the intriguing and marked systemic effect evoked by the endurance exercise. Although the changes in cluster 1 mostly disappeared 90 min after cessation of exercise, aforementioned transcription factors set whole gene networks into motion. At later time points, these can be translated into increases in many genes that are typically responsible for muscle adaptation to endurance exercise such as *PPARGC1A* (PGC-1α, *VEGFA* or *TRIM63* (MuRF-1), noted in cluster 2, which importantly also support our PCR-data. Other highlighted changes in gene expression that were increased immediately after the ER-Arm protocol, were genes usually favored by endurance exercise and involved in oxidative phosphorylation and fuel utilization. Finally, it must be noted that firm conclusions cannot be made from the global transcriptomic data given the reduced number of subjects, however, these data may be of value for how to target future analysis in studies on, for example, exercise induced molecular cross-talk or the effects of exerkines.

On the other hand, we found that the resistance exercise induced gene programs mostly associated with repair and maintenance, such as extracellular matrix remodeling, collagen synthesis or muscle development (clusters 5 and 6) were significantly attenuated when the arm resistance exercise was preceded by leg endurance exercise. This supports the notion that the interval endurance exercise induced systemic changes to transcription programs. Although the potential mechanism remains to be elucidated, one candidate could be the mechanical component brought about by increases in blood flow and pressure, with possible interaction from muscle-secreted active molecules.

It could be argued that the potentiated effect on PGC-1α and MuRF-1 gene expression, as well as the effects noted for global transcription, in the ER-Arm trial was mediated through molecular changes due to activation of the triceps muscle during interval cycling rather than a systemic effect. This could maybe have been clarified by taking an additional biopsy following interval cycling, however, considering the small size of the triceps muscle, and the already large number of more prioritized samples, this was not done here. In the aspect of gene expression, an immediate post interval cycling biopsy would also be of minor value as notable changes are not detectable until hours after exercise. In addition, the activity of the arm muscles during interval cycling is likely to be small since the stabilizing action of the arm muscles are minor during stationary indoor cycling and may therefore not have a significant impact.

In conclusion, when resistance arm exercise was preceded by interval endurance exercise with the legs, mRNA transcripts and proteins involved in both anabolic and catabolic processes were significantly altered. The larger increases in the levels of PGC-1α and MuRF-1 mRNA in the ER-Arm trial are indicative of a systemic effect. This was also supported by the exploratory RNA sequencing showing a large number of genes differently expressed in the ER-Arm trial compared to the R-Arm trial. Preceding interval cycling exercise attenuated the rise in S6K1 and 4E-BP1 activation observed in the triceps muscle immediately after the resistance exercise, but this effect disappeared rapidly and did not involve other mTORC1 signaling proteins or detectably affect the rate of protein synthesis. Our present observations indicate that alterations in systemic factors in response to interval leg cycling can influence the expression of genes and proteins involved in the adaptive remodeling of arm muscle induced by resistance exercise. Collectively, these findings argue that training involving both modes of exercise together might be advantageous. The factors involved in and mechanisms underlying these potential systemic effects remain to be unravelled.

## Supplementary Information

Supplementary Information.
