# Supplementary material for: High-intensity leg cycling alters the molecular response to resistance exercise in the arm muscles
Source: Sci Rep. 2021 Mar 19;11:6453. doi: 10.1038/s41598-021-85733-1 (PMC7979871; doi:10.1038/s41598-021-85733-1)
Supplement: Supplementary file 1 — Supplementary Information. [file 41598_2021_85733_MOESM1_ESM.pdf]

SUPPLEMENTARY INFORMATION

High-intensity leg cycling alters the molecular response to resistance exercise in the arm muscles

Marcus Moberg[1], William Apró[1,2], Igor Cervenka[3], Björn Ekblom[1], Gerrit van Hall[4,5], Hans-Christer Holmberg[3], Jorge L Ruas[3] and Eva Blomstrand[1,3]

[1]Department of Physiology, Nutrition and Biomechanics, Swedish School of Sport and Health Sciences, Stockholm, Sweden. [2]Department of Clinical Science, Intervention and Technology, Karolinska Institutet, Stockholm, Sweden. [3]Department of Physiology and Pharmacology, Karolinska Institutet, Stockholm, Sweden. [4]Department of Biomedical Sciences, Faculty of Health and Medical Sciences, University of Copenhagen, Denmark and [5]Clinical Metabolomics Core Facility, Clinical Biochemistry, Rigshospitalet, Copenhagen, Denmark

Corresponding author:

Marcus Moberg, PhD

The Swedish School of Sport and Health Sciences

Box 5626, SE-114 86 Stockholm, Sweden

E-mail: marcus.moberg@gih.se

SUPPLEMENTARY FIGURE S1

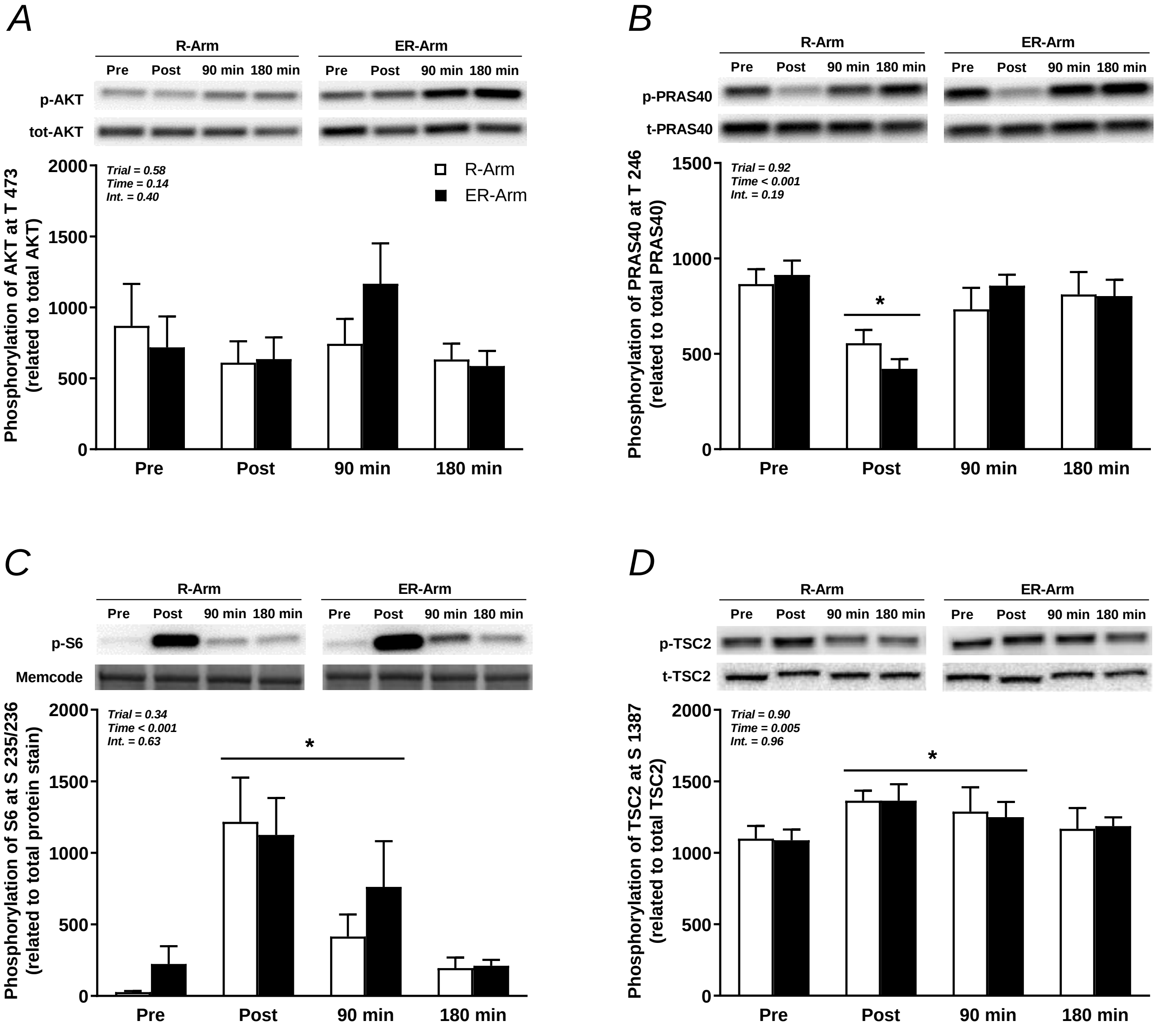

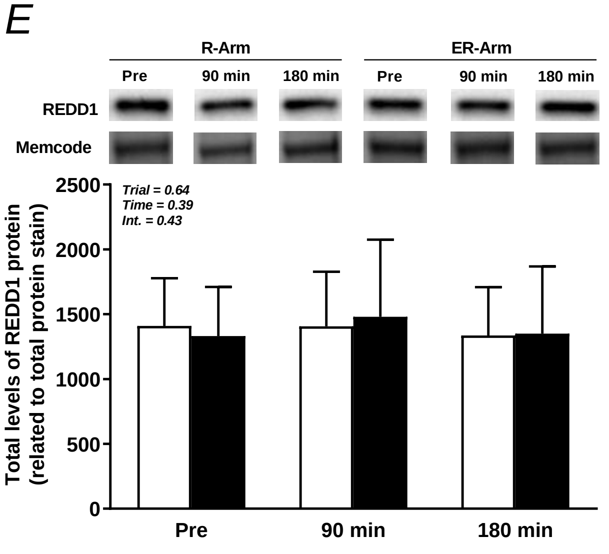

Supplementary figure S1. Phosphorylation of AKT at Thr473 (A), PRAS40 at Thr246 (B), S6 at Ser235/236 (C), TSC2 at Ser1387 (D), and levels of REDD1 protein (E) at different stages of both trials. The values presented are means ± SD for 8 subjects. *P < 0.05 vs. Rest. Symbols denoted with a line represent a main effect. Representative immunoblots (from strips of full-length blots) of both phosphorylated (upper panel, expect for REDD1) and total protein or total protein stain (lower panel) from one subject are shown above each graph. The two sets of bands have been rearranged, and separated with a white space, to fit the order of the trials in the graphs. Pre = baseline, Post = immediately after exercise, 90 and 180 min = length of recovery.

SUPPLEMENTARY FIGURE S2

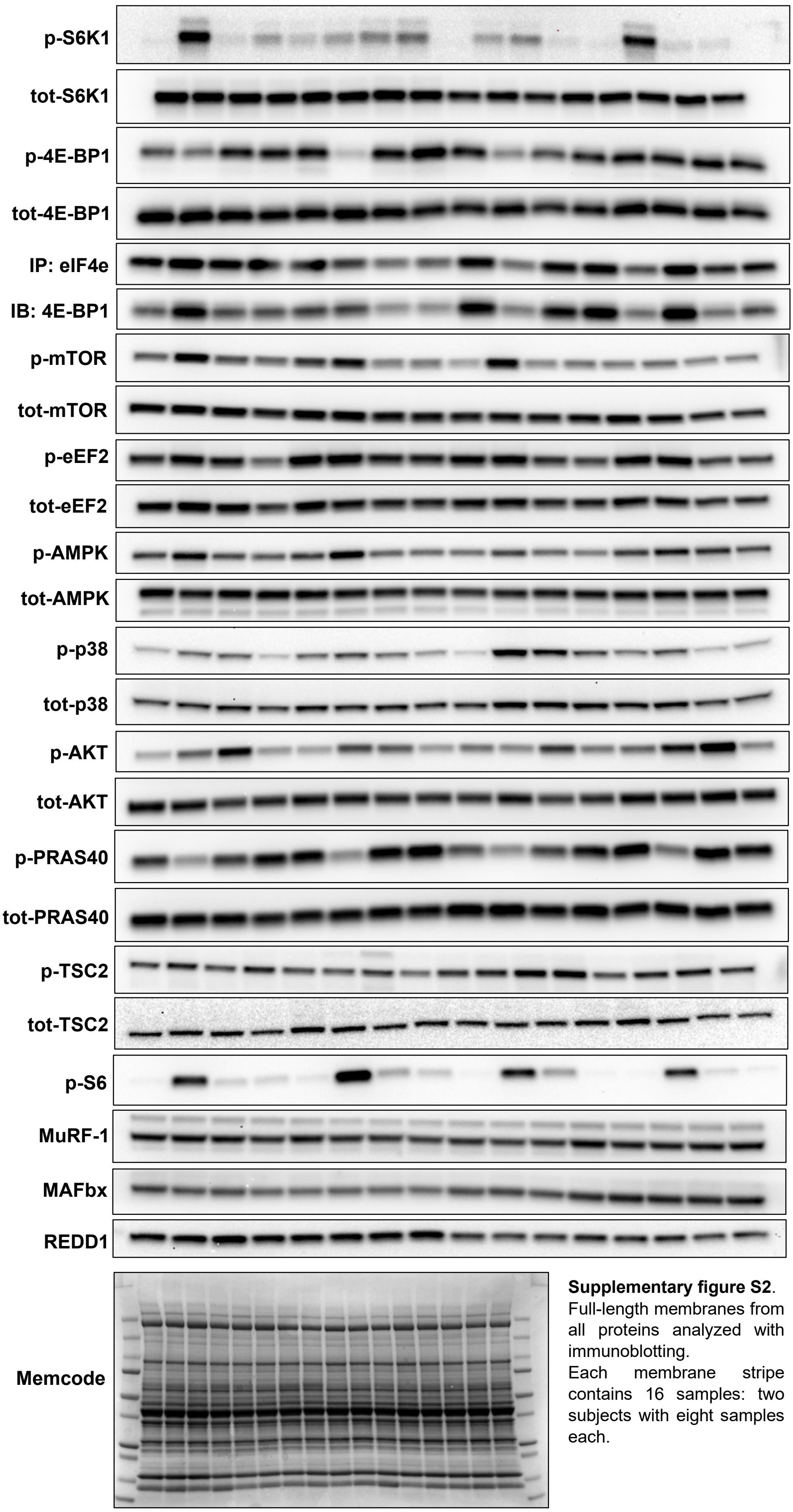

Supplementary figure S2. Full-length membranes from all proteins analyzed with immunoblotting.
Each membrane stripe contains 16 samples: two subjects with eight samples each.
